# Ethical conflicts experienced by intensive care unit health professionals in a regional hospital, Limpopo province, South Africa

**DOI:** 10.4102/hsag.v25i0.1183

**Published:** 2020-04-16

**Authors:** Dorah U. Ramathuba, Hulisani Ndou

**Affiliations:** 1 Department of Advanced Nursing Science, University of Venda, Thohoyandou, South Africa

**Keywords:** conflicts, ethics, health professionals, intensive care unit, ethical frameworks, professional bodies

## Abstract

**Background:**

Conflicts arise when healthcare providers disagree about providing optimal care to critically ill patients where resources and services are constrained.

**Aim:**

This study investigated ethical conflicts experienced by intensive care unit (ICU) healthcare professionals working in a regional hospital, Limpopo province of South Africa.

**Setting:**

The study was conducted at a rural public regional hospital in Vhembe district, Limpopo Province. Communities served by the hospital are poor and medically uninsured.

**Methods:**

This study adopted a qualitative, exploratory and descriptive design. The target population comprised Health care professionals working in an ICU of the regional hospital. Purposive sample was selected and 17 unstructured interviews were conducted. Tesch’s method of data analysis was used. Ethical considerations were adhered to.

**Results:**

Patients’ care needs were compromised because of the unavailability of beds and high-technology equipment, such as well-functioning ventilators. Doctors were not having the necessary skills required in the ICU as the majority were on community service/internship and nurses acted beyond their scope of practice because of a lack of adequately trained intensive care specialists. Infection control practices were overlooked and ‘use once’ pieces of equipment were reused. Conflicting values between nurses, patients and family of patients exist.

**Conclusion:**

Lack of resources compromises provision of optimal and intensive care. Patients were prone to infections and their safety might have been compromised.

## Introduction

South African healthcare systems have experienced tremendous changes since the dawn of democracy, and access to healthcare at all levels of care has increased ethical conflicts among health professionals. Communities are aware of their needs and rights when visiting health services and institutions are overwhelmed in an effort to satisfy consumer demands. The private healthcare system is not affordable by the majority of the population, leaving the public health services challenged to provide just and equitable healthcare services in accordance with the *Constitution of the Republic of South Africa* (Act no. 108 of 1996). The complexities in public healthcare services require adherence to ethical principles in serving communities. The Hippocrates oath taken by doctors, and the nurses’ oath affirms the intentions of providing a just and equitable service. However, because of sociopolitical factors, public health services in rural communities are still faced with dire shortages of resources which increase moral distress among healthcare professionals (Ulrich et al. 2010). The majority of the population cannot afford private healthcare services, and the intensive care units (ICUs) in regional hospitals are strained.

In most instances, health professionals in healthcare settings are under duress and are continually solving problems and dealing with conflict situations (Kälvermark et al. 2004). Health services face tremendous challenges because of changing landscape in social, political, legal and organisational factors resulting in ethical conflicts, and oftentimes policies fail to address such conflicts.

The ethical problems cannot be adequately managed with policies designed to curb the situation in health settings. Ethical conflicts among health professions are varied because of the professional competencies required in patient care in healthcare settings. Intensive care unit health professionals are exposed to ethical conflicts on a daily basis. Falcó-Peguerolesa et al. (2016) indicated that healthcare professionals face different types of ethical dilemmas such as interpersonal conflicts with patients, families and other colleagues in relation to informed consent, confidentiality, treatments and medical procedures. Previously nurses waited for directions from doctors, whereas presently nurses have reclaimed their role in practice, and they are able to design care plans and patients’ protocols and advocate for patients in their care (Wilson-Barnette 1986). These ethical dilemmas cause health professionals to suffer from stress. Kälvermark et al. (2004) identified several stressors such as increased workload, poor morale and poor job descriptions. Stress in the workplaces causes decreased morale, psychological and emotional distress among health professionals. Ong, Yee and Lee (2012) reported that moral distress manifests itself with negative behavioural characteristic and burnout among health professionals. Moral distress is associated with emotional exhaustion. Kälvermark et al. (2004) outline the major ethical conflicts:

*Moral uncertainty* arises when one is not sure whether there is an ethical dilemma, or when one assumes that there is an ethical dilemma but is not sure what principles or values apply in the ethical conflict. (p. 1077)

*Moral dilemmas* occur when principles or values conflict and there are good reasons to support mutually inconsistent courses of action. *Moral distress* ‘occurs when one believes and knows what is morally right, but institutional constraints make it difficult to do the right thing’. Wood (2014) indicates that when circumstances force health professionals not to practise what is expected of them, it results in moral distress and employee turnover.

## Purpose

The aim of this study was to investigate the ethical conflicts experienced by ICU healthcare professionals in a regional hospital in Limpopo province of South Africa.

## Objectives

The objective of this study was to explore and describe the types of ethical conflicts occurring in a regional hospital in Limpopo province of South Africa.

## Methodology

A qualitative research design, following the phenomenological approach, was used to explore and describe the ethical conflicts experienced by ICU health professionals in a regional hospital of Limpopo province. The design was appropriate to answer the research question because it emphasises on understanding the human experiences as it is lived.

## Research setting

The project was conducted in Vhembe district of Limpopo province, in an ICU of regional hospital, which serves six district hospitals that transfer critical and high-care patients in need of intensive care.

## Population and sampling

The population was all health professionals working in an ICU of a regional hospital in Vhembe district, Limpopo province.

Non-probability purposive sampling was used to sample doctors and nurses who were working in ICU for a period of 1 year and have consented to participate in this study. Purposive sampling is a judgemental sampling method which the researcher selects participants who best represent the population under study and are informative about the phenomenon being studied.

## Data collection

To have an in-depth understanding of the health professionals’ experiences, a face-to-face unstructured interview was used, guided by the central question: ‘what are the ethical conflicts you experience in ICU on a daily basis?’ Using probing, paraphrasing and clarification statements allowed eliciting of thick description and exploration of the phenomenon. Furthermore, field notes were also recorded because they assist in capturing the observations in the research setting and are able to give interpretation or meaning (Polit & Beck 2016). Data collection took place from April 2017 to July 2017 with 17 health professionals. The participants were informed about the study purpose and the use of audio-tape and writing of field notes and were not coerced to participate. During the data collection process, the participants’ names were not captured and all interview schedules were securely kept for anonymity and confidentiality.

## Data analysis

Data analysis started during data collection. The audio recordings and field notes were transcribed verbatim and return visits were made to verify with participants if what has been captured is indeed the correct information. The transcripts were numbered and assigned letter D (doctor) or N (nurse) to represent professional status to ensure the interviewee confidentiality and anonymity. Data were analysed inductively and deductively with formation of themes (Creswell 2013) (see Table 1).

**TABLE 1:** Measures for ensuring trustworthiness.

Strategy	Criteria	Applicability
Credibility/truth value	Prolonged engagement	The second author worked with participants as an ICU nurse. The researchers are known in the hospital as part of service delivery and the researcher stayed in the field for an extended period of time to build trust and rapport with participants
Triangulation	Multiple investigators Multiple source of data Member checks Peer evaluation	The supervisor participated in all phases of the research conceptualisation until the final report. Interviews, audio recording and field notes were used during data collection to ensure that observations and experiences are captured. Follow-up interviews were done with professional nurses and doctors validate data that were already gathered. The research was discussed with other supervisors in the faculty who are experienced in qualitative research.
Applicability and transferability	Interview techniques Sample Dense description	Central question used during data collection opened communication. Interview technique such as repeating, rephrasing, paraphrasing increased credibility of findings. A purposive sampling method was used to nominate knowledgeable participants who were involved in the experience of ethical conflicts. Demographic information of participants was provided. Detailed information about the context/study setting, participants and methodology was provided and will allow other researchers to transfer findings. Reporting the actual situations that have been investigated and, to an extent, the contexts that surround them increases insights to the reader to employ the method and embrace the real situation. The study findings were contextualised and supported with literature control.
Consistency and dependability	Code–recode Dependability audit Dense description of research methods	Data were analysed and the supervisor verified the data and themes and sub-themes for consistency. The verbatim transcripts, audio-tapes and field notes were given to the independent coder, thereafter findings were compared for similarities or differences and consensus was reached with agreement. Triangulation of face-to-face interviews, audiotape and field notes made auditing possible. Full description of the operational details of data gathering, addressing the process of what was done in the field, use of communication skills and analysis methods, the reporting of verbatim results makes the findings dependable.
Confirmability	Neutrality Confirmability audit	The researcher remained neutral in her perspective and decreased her distance between the participants by not being emotional during prolonged engagement. The supervisor also listened to the tapes to verify interpretation and conclusions. A meeting was held with an independent coder and supervisor to compare and verify findings and reached consensus by agreement.

Note: Measures to ensure trustworthiness were applied according to Guba and Lincoln (1985) citing four aspects, which are credibility, transferability, dependability and conformability.

ICU, intensive care unit.

### Ethical consideration

Ethical clearance to conduct the study was obtained from the University Ethics Committee (SHS/16/PDC/32/3008) and the Provincial Department of Health of Limpopo province.

## Findings

### Demographic data

Seventeen participants consisting of seven doctors and 10 nurses composed the sample for this study. Seven professional nurses had more than 11 years of working experience as ICU nurses and only three had less than 10 years of experience. Out of the seven doctors, four had 5 years of experience post-internship and only three had less than 5 years of experience.

#### Theme 1: Inability to provide inpatients needs because of lack of resources

Health professionals’ role is to provide care; however, at times they are unable to provide due care because of hospital practices and other reasons such as patients being too sick to benefit from the ICU and/or bed shortages. Only one sub-theme emerged from this theme.

**Sub-theme 1.1: Lack of intensive care unit beds impacting provision of care:** The participants in this study experienced a dilemma in provision of care because of limited resources when all the patients were in need of care. Participants stated: participant no. 11 (N) pointed out that:

‘[*W*]e quite often we are faced with the situation where we see a patient and we need to decide, based not only on the condition of the patient but on the availability of ICU beds. Whether we are going to take this patient to ICU or not. This is an ethical dilemma more than a medical dilemma because medically speaking you’ve got no doubt that this patient needs ICU but let’s say you’ve got one ICU bed and you’ve got three patients who need ICU. You cannot admit all of them because you don’t have beds.’

Participant number. 13 (D) stated that: ‘Because of our resource limitation, as a doctor I am supposed to choose who needs ICU now as compared to one another’ ‘Choosing is like playing God’.

Because of rapid population growth, hospital capacity has been an issue that needs to be addressed to ensure that the people received the necessary service and access to healthcare. This can be possible only if the department of health invests on infrastructure and acquires more hospital beds in public hospitals, so as not to cause mental distress to healthcare professionals. Bucci et al. (2016) also indicated that emergency departments (EDs) worldwide face challenges of crowding, cost containment and excessive waiting times because of shortages of hospital beds.

#### Theme 2: Compromised quality care because of inequity in allocation of resources

South African health system is still trying to restructure and revitalise itself, and the public health system is burdened by poverty and unemployment because the majority of population uses the public health system and its budget is through provinces. This theme had two sub-themes.

**Sub-theme 2.1: Unfair allocation of resources for provision of patient care:** Participants expressed their concern regarding allocation of resources. Public hospitals are allocated budgets to cater for needs of communities or patients being served; however, guidelines or directives within the health system may impact on resource allocation. Participants had this to say: participant number 7 (N) mentioned that: ‘the worst is to ventilate the patient without the blood gas machine’.

Participants number 9 (D) mentioned: ‘The lack of functional ventilators at all times is also a problem’.

Participants number 2 (N) said that:

‘More than being a dilemma I think it is a political problem, because we are regarded as people who are in the rural, and we usually do not get equipment or whatever item that is used in ICU. We are categorised as people who are not supposed to get certain things. I will just make an example of a T-piece. A T-piece is a small connection for weaning patient from ventilator to oxygen, but you will receive information from pharmacy that it is a tertiary item, a motivation is required.’

Participant number 4 (N) indicated that: ‘Sometimes you will find that the monitor that the patient is using does not show saturation. For you to monitor the patient’s saturation patients have to share the oximeter’. Furthermore, participant number 5 (N) raised her concerns stating that:

‘sometimes we are forced to use the same suction catheter for the whole day because they are out of stock. If I discard it with every suctioning tomorrow, there will be no suction catheter to suction at all.’

The above narratives are depressing as they affected the participants morally because they wanted to provide due care to patients and need the required resources to provide quality care. Decisions relating to the allocation of healthcare resources have been inevitable, either between different competing services and interventions (i.e. priority setting) or across different patients (i.e. rationing). However, the methodological approach is in view of allocating resources in an efficient and fair way (Angelis, Kanavos & Montbeller 2017).

**Sub-theme 2.2: Lack of human resources resulting in moral duress:** Participants expressed that it is difficult to provide care in the ICU as they have to take detrimental actions of working outside their scope of practice for continuity of care, by overlapping their roles where they thought it was necessary and it sometimes strained relations of the team as some doctors may not be happy to endorse what has already being implemented. Participants explained their dilemma in this way:

Participant 17 (D) mentioned:

‘[*S*]ometimes as a doctor I have to come and endorse something that nurses have started or put, may be you are not 100% that you really wanted to do this but, because it has already been done, then you endorse it.’

Participant 3 (N) mentioned:

‘Nurses are supposed to be given orders by a doctor but in our case it is vice versa. Instead of helping us, we end up helping them. Most of the decisions are taken by the nurses and the doctor just endorse.’

Participant number 4 (N) indicated that:

‘[*T*]he allocation of our ICU leaves us with no choice but to use lower categories nurses like staff nurses to nurse critically ill patients. It is a dilemma because I don’t have another staff, I only have those.’

Miseda et al. (2017) indicated that skill gaps on selected specialists (healthcare professional whose practice is limited to a particular area, such as a branch of medicine, surgery or nursing), especially, are limited by their professional bodies to adhere to a particular scope by virtue of certification or qualification. Furthermore, human resource shortages have constrained the achievement of health-related development goals and impede progress of universal health coverage particularly in specialised healthcare.

#### Theme 3: Concerns regarding maintenance of professional boundaries and confidentiality during communication with patients’ relatives

Safeguarding patients’ rights, with respect to an individual’s personal health information, is an ethical and legal obligation of healthcare providers. Poor infrastructure can also pose problems to communication as there can be no office space or private room for counselling and discussing patients’ health outcomes as multidisciplinary team.

**Sub-theme 3.1: Privacy and confidentiality conflicting with the perceived need for information:** Participants indicated that it was important to assist the patient to recuperate; however, it became difficult when the patients’ condition had deteriorated and it was difficult to break through to get information while maintaining professional secrecy. Participants expressed their concerns in this way.

Participant number 14 (D) said: ‘There is no place called away from the patients where patients can be discussed because the room is so small’. There is no counselling room where relatives can be interviewed. ‘Relatives are interviewed in front of other patients’.

Another participant indicated that at times the patient had not confided with relatives about their health and treatments and as a nurse you must still respect the decision of the patient amidst their condition.

Participant 5 (N) said:

‘It is difficult to maintain confidentiality in ICU, especially when you are dealing with unconscious patients. There are patients who can take chronic medication for years without anyone in the family knowing, so when you start asking about his chronic medication the relatives become surprised.’

It is the duty of health professionals to protect the well-being of those entrusted to their care and always keep the patient’s best interest in mind when communicating with family members. Protecting the integrity of the nurse–patient relationship and patient rights is a sacred trust.

**Sub-theme 3.2: Moral distress related to beneficence:** An integral part of health professionals’ work is the foundational ethic of beneficence, which compels striving for greater good. Participants experienced distress when making decisions regarding provision of care by doing what is good and right for the patients. A participant indicated (Participant 3 [N]) that

‘One day we have to admit the second burns patient. The doctors tried to transfer the patient to other hospitals and there were no beds available. We were forced to nurse that burns patient in the main ward because the separate room was already having a burns patient. Can you imagine, nursing sixty percent burns in a ward where everyone just walks in?’

Maintaining good hygienic standards and maintaining isolation technique are what is good and of benefit for the patient towards recovery. Another participant showed guilt feeling and said (Participant 10 [D]):

‘The lack of high care unit is a big dilemma for us, because there are some patients who after being stabilized in ICU could be transferred to high care, but because there is no high care. We keep them there longer, they occupy the bed, I know that if I move this one to the general ward is going to die. Sometimes you make those decisions based on knowing that if I move this patient now, there is one patient who needs the bed more than this one but you can’t move this one because in the ward is going to die anyway.’

It is always fulfilling to observe a patient who had progressed from the ICU, and you still wish what is best for the patient when the patient is transferred out to step down unit/facility.

The ethical principle of beneficence requires healthcare professionals to treat their patients in a way that provides maximum benefit to that patient, meaning that it is the responsibility of the healthcare professionals to protect their patients. Bhanji (2013) indicates that beneficence goes along with the ethical theory of utilitarianism which believes in the actions that provide the highest good for the maximum number of people.

#### Theme 4: Protecting patient autonomy

A patient’s independence is traditionally the highest priority in bioethics and emphasis is intended to prevent patients from being coerced, especially by medical professionals.

**Sub-theme 4.1: Preserving right of self-determination and maintaining trust relationship with the patient:** Participants indicated that although they wanted to assist the relatives but felt indebted to the patient, they did not want to compromise the nurse–patient relationship and trust because the patient made a decision when he was capable.

Participant number 17 (D) indicates that:

‘There was a patient who was in acute renal failure, he needed dialysis. Staff at a receiving hospital wanted to know the Human Immune virus (HIV) status because they need to decide which machine to put the patient on. When the patient was still talking before he went into coma, he totally refused to be tested for HIV. Now that the patient is in coma the relatives say they can give consent to test for HIV. Our hands were tied. The relatives cannot supersede the will of the patients.’

Respecting the patient’s autonomy and right to self-determination is important in healthcare; however, above all we must do good than harm, options need to be weighed. Bester, Cole and Kodish (2016) indicate that a substituted judgement obviates the need for including other measures, such as weighing the welfare of the patient. Surrogate decision-making moves away from the ideal of informed consent towards valorising protection of the patient, and in such situations informed consent is not feasible, given the primacy of the ethical obligation to do no harm, a clinician should focus on her obligation to protect her patient rather than fixating on informed consent.

#### Theme 5: Conflicting beliefs

In practice, conflicts with moral uncertainty arise between the principles of autonomy and beneficence or justice, between duties to the patient and one’s duties to one’s profession, and with families or patients’ loved ones on what is ‘good’ for a particular patient.

**Sub-theme 5.1: Moral uncertainty pertaining to beneficence and non-maleficence (doing good and protecting harm):** Participants experienced moral conflict in what was good for the patient pertaining to their right to autonomy and justice, and uncertainty is also brought about lack of factual knowledge about other indigenous practices. One participant indicated that:

Participant 12 (N) indicated that: ‘There was a patient who was refusing amputation and she will only scream while in pain to say amputate this limb.’

Patients should make decisions when they are capable and in the right state of mind. The principle of proportionality means that the minimum amount of medication should be used in order to achieve the desired effect of pain relief; if medication is increased until pain is controlled, the patient may be able to make a competent decision.

Family members are also a source of conflict in health settings as they sometimes impose their values and beliefs in the care of their patient. In Western cultural values, the value of respect for autonomy is mostly tied to human beings as individuals while other African societies and ethnic minorities place value on family and community. Relationship ethics or ethics of care views commitments and relationships to others as the basis of ethical life.

Participant 13 (N) said: ‘There was another lady, who refused to be transfused blood because of her religion and she had bled a lot during operation, she was very anemic’.

Participants 17 (D) had this to say: ‘You know when a patient is very ill, and you know you have got nothing more to offer, then the relatives come with their own things. And you are sure this patient is not going to make it. Do you allow them or not? Sometimes they ask to bring some muti/herbs for the patient, what should we do, do we allow them?’

Participant 8 (D) added: ‘On other occasion we have come across a situation where the relative even asked for the critically ill patients. They say they wanted to explore other indigenous alternatives’.

Hoop et al. (2008) indicate that the use of native and traditional remedies has been considered unethical, based on the belief that these treatments were unproven and unprofessional; however, as medicine has become more culturally pluralistic, the ethics of using traditional medicines has developed and physicians are legally and ethically required to support patients’ decisions to use the remedy if there is medical evidence that the treatment is safe or ineffective, clinicians have a duty to inform patients, clinicians should safeguard patient well-being to the best of their ability.

## Discussion

Intensive care units have advanced complex technology to support critically ill patients in healthcare settings; however, the hospital environment can be distressing to patients, relatives and health professionals when tension arises in the process of providing care. Ethical dilemmas occur in healthcare settings as a result of conflict in values and principles that arises when there are decisions that need to be taken and the responses to decisions or solutions cause ethical tension and duress when health professionals have to choose between two options and the choice may have desirable and undesirable results (Pacsi 2008; Scanlon & Murphy 2014). Furthermore, Fant (2012) alludes that ethical dilemmas can be a result of clashes of moral principles, the healthcare professionals’ values and beliefs in contrast to that of the patients.

The findings revealed that health professionals were unable to provide quality patient care because of limited resources such as availability of beds and some of the equipment which comprised the quality of care. Work environments should be provided with necessary equipment to provide efficient patient care. Shortage of resources can cause stressful situations for health professionals, and they suffer emotional stress/duress when they are unable to help the patients because of administrative constraints. Resource allocation should be distributed fairly to all health settings to prevent compromising provision of care, and the principle of equity and justice should prevail in healthcare settings because health professionals have the duty to provide care. Naidoo, Singh and Lalloo (2013) indicated that in South Africa there is a criterion for placing patients in ICU which is bed availability and the patient’s previous and present condition. It is problematic because health professionals may be biased in rationing limited resources among patients and ethical dilemma arises between beneficence and providing justice. Häggström, Mbusa and Wadensten (2008), in their study in Tanzania, reported that often the patient’s autonomy is compromised because of financial constraints or inability to pay for services. This is the case in rural public ICUs because the majority of the population is from low socio-economic standing and resources are not equitably distributed.

de Beer, Brysiewicz and Bhengu (2011) assert that ICUs are structured and graded in level IV units are high-dependency units. The first level is affiliated to teaching in universities while the fourth level is high-dependency units care, implying that ICUs in public rural hospitals can be regarded as high-dependency units care or graded as such because they lack most of the basic resources like a well-functioning ventilators, nurses compromised patients’ lives by alternating patients on a ventilator which compromises the right to patient safety. Krishnamoorthy, Vavilala and Mock (2014) reported that ventilators are necessary as they save lives; however, they require intense maintenance. The findings revealed that the very ventilator was long serviced, and for proper patient care, blood gas machine should be available to monitor the oxygen status, which was not the case. It is important for procurement and management to provide basic equipment used in the ICU and adhere to the manufacturer’s instructions on equipment handling and care for efficiency and effectiveness of its functioning.

Shortage of skilled staff was also highlighted as a dilemma and resulted in moral duress. Intensive care unit uses high technology and requires staff who are competent, and there was no intensivist or a specific doctor assigned for emergencies and only few ICU-trained registered nurses who delegated junior staff under their supervision on issues beyond the scope of practice. Nurses also experienced relationship problems with the doctors in cases where they took initiative of doing what they regard as morally good for the patients, advocating and serving what is best for the interest of the patient. Kälvermark et al. (2004) indicate that hierarchical structures between different professionals have a negative impact on how a professional can act out their own moral position. Sometimes health professionals in junior ranks in the hierarchy experience moral stress when they have to carry out orders from a superior against their own conviction.

Furthermore, Ulrich et al. (2010) reported that respondents in their study cited protection of patient rights as their most frequent ethical issue, and staffing inadequacies were the most stressful issue. The authors further indicate that without sufficient staffing, it is difficult to meet the ethical standards of professional practice such as protecting the rights of individual patients and families, alleviation of sufferings and preserving their own integrity.

Wallace et al. (2012) indicate that a 24-h intensivist may provide instant and timely treatment and care, even during emergency. Findings indicate that doctors who were working in ICUs were also working in other wards and came only once in a day to see patients and in case of emergency, which led to registered nurses making some changes before the doctor arrived, such as changing types of intravenous fluids or withholding administration of medication before the doctor arrives because at times it becomes difficult to get hold of the doctor on the phone and thus resort to their therapeutic skills. Protecting the rights of the patient is a basic ethical tenant, and professional nurses experienced moral uncertainty regarding professional advocacy, by choosing to do what they think is morally good at the time to benefit the patient or either to withhold treatment in order not to inflict harm. Registered enrolled nurses were also delegated under supervision at times in providing care to critically ill patients. Matlakala, Bezuidenhout and Botha (2014) and Bhagwanjee and Scribante (2008) allude that ICU requires competent and skilled healthcare professionals for safe and effective intensive care practice.

Health professionals are always having the patients’ best interest at heart. The paternalistic model of decision making considers beneficence and non-maleficence ahead of patient autonomy; therefore, nurses and doctors are morally obliged not to cause harm but do what is good for the patient. However, lack of proper infrastructure resulted in premature discharge of patients to the general wards where care is suboptimal and could have negative outcomes, unlike transferring the patient to high care. Furthermore, the infrastructure compromised privacy and confidentiality as the ward is just an open space usually divided by curtains. Information privacy is also compromised during counselling, report taking and sharing of information because of infrastructure challenges where patients were not separated by age such as paediatrics and adult units, as in the case with the burnt patient who could not be isolated because of limited space and compromising on infection control. Wenham and Pittard (2009) state that a single room for each patient should be the long-term strategy as this preserves privacy, dignity and confidentiality and may promote issues related to infection control. Furthermore, Facility Guideline Institute (2014) suggests that providing the patient with privacy and dignity is essential when designing critical care units as most patients are vulnerable and unable to communicate their needs.

Communication is important between members of the health team and family members. Families are usually under duress if they realise that communication is not forthcoming, or less information is provided. Personal information of the patient should remain private and confidential. Participants in this study also felt that the prognosis of the patient was poor, there was nothing more to offer the patient and could not tell the relatives or allow them to resort to other indigenous ways in order to save them the distress. Autonomy encompasses veracity/truth telling so that relatives or families may be able to make an informed decision; however, the conflict arises on how to communicate such information about their loved ones. Sundean and McGraths (2013) indicate that providing families and relatives with knowledge about the condition and prognosis of the patient demonstrate respect for autonomy.

Farahani et al. (2014) and O’Kelly, Urch and Brown (2011) indicate that nurses and doctors must weigh options in truth giving and provision of hope as it differs across cultures and worldwide. Truth telling differs in developed and developing countries and cultural background of societies. Participants in this study could not reveal reasons why the patient was not transferred because of HIV tests because the patient refused to be tested and allowing relatives to consent for patient would be compromising the patients’ confidentiality, privacy and respect for the patient.

Communities in rural villages of Limpopo have the different belief system according to ethnicity. And health professionals are aware of some of these practices because they are from similar backgrounds; however, professionally it becomes an ethical conflict of not respecting the patients’ decisions and culture. Moral uncertainty often prevails when health professionals have to weigh the ethical principle of not doing harm and what will benefit the patient.

Non-maleficence means non-harming or inflicting the least harm for the benefit of the patient. In determining the patients’ best interests (beneficence), health professionals are obliged to consider the views of the family. Furthermore, the family members can effectively advocate for their loved ones, and also represent the patient’s personal and cultural values even if they are not legally appointed surrogates (Modra & Hilton 2015).

Previously healthcare professionals were unilaterally taking decisions on behalf of patients based on their own professional values and understanding. Patients and relatives are now concerned with the health conditions of their loved ones and need to be involved in the process of care. Ong et al. (2012) also concur that respecting the patients’ autonomy and decision-making is important in minimising ethical conflicts, indicating that patients are no longer passive and they should be involved in their care.

Participants in this study indicated that the patient who was suggested amputation agreed only when in pain. Similar situations were observed in this study where relatives wanted to give consent on behalf of the patient when the patient was unconscious, the patient had initially refused to consent when she or he was able to. Furthermore, Moodley (2011) reported that conflict arises when a patient refuses treatment and doctors need to provide treatment, like a patient refusing to get blood transfusion. Beneficence guides healthcare providers to respect the patient’s autonomy by recommending alternative treatment options and respecting the patient’s beliefs even though if it does not coincide with the healthcare provider’s values (Sundean & McGrath 2013).

As health professionals, it is their duty to provide information and also respect patients’ decision. However the principle of autonomy is not final, but rather *prima facie*, meaning that it can be overruled to benefit patients’ right to life.

## Limitations

This study was based at a regional hospital in one province, which makes it difficult to generalise the findings.

## Recommendations

Healthcare settings, especially public hospitals, must provide better support resources and structures to decrease moral distress.

A need for further education in ethics to help professionals to understand better their own process of ethical decision-making and create a greater readiness for related situations and patient care. Fostering ethical cultures may help to address the root causes of moral distress.

Family and patient education are important in maintaining nurse–patient or family relationship.

## Conclusion

Ethical conflicts are a daily occurrence in healthcare settings especially in high care and ICUs. Health professionals face these challenges of lack of resources, poor communications between staff and family and poor decision-making in poor resource settings, resulting in moral distress and depersonalisation. Interventions to address these challenges are necessary in healthcare setting by provision of resources and building capacity of the work force in ICUs.
